# Seasonality Has Greater Influence on Amphibian Cutaneous Mycobiome than Host Species

**DOI:** 10.3390/jof11070473

**Published:** 2025-06-22

**Authors:** Han Zhang, Kunyang Zhang, Hongying Ma, Jie Deng, Cheng Fang, Hu Zhao, Xiaoran An, Jianlu Zhang, Qijun Wang, Wei Jiang, Fei Kong

**Affiliations:** 1 Shaanxi Key Laboratory of Qinling Ecological Security, Shaanxi Institute of Zoology, Xi’an 710032, China; hanhanr9@163.com (H.Z.); mhying7916@163.com (H.M.); dengjie0311@ms.xab.ac.cn (J.D.); f-chin@163.com (C.F.); zhaohu2007@126.com (H.Z.); zhang-jianlu@ms.xab.ac.cn (J.Z.); wqjab1@126.com (Q.W.); jiangwei197981@163.com (W.J.); 2 College of Animal Science and Technology, Northwest A&F University, Yangling 712100, China; xoozhan@nwafu.edu.cn; 3 Fisheries Research & Technology Extension Center of Shaanxi, Xi’an 710086, China; ax121121@163.com

**Keywords:** Qinling Mountains, amphibians, skin, mycobiomes, seasonal variation, host species difference

## Abstract

Amphibians face severe threats from chytridiomycosis, and their skin microbiota plays a crucial role in pathogen defense. However, studies on their mycobiomes are limited. We hypothesized that amphibian cutaneous mycobiomes vary with seasonal variations and host species. To test this hypothesis, we used internal transcribed spacer (ITS) amplicon sequencing to identify the cutaneous fungal communities of two frogs from the Qinling Mountains of China, namely *Pelophylax nigromaculatus* and *Nanorana quadranus*. We also compared our ITS amplicon data with those of 30 known anti-Bd fungal ITS sequences to identify Bd-inhibiting fungi in the samples. The results showed that seasonal variation exerted a significantly stronger influence than host species on the fungal community structure (alpha diversity, beta diversity, species composition, abundance, and biomarkers). In the fungal community composition, intergroup consistency was significantly higher at the phylum level than at the genus level; however, one unidentified genus was present in samples from both frogs from different seasons. Anti-Bd fungi were detected in the skin fungal communities of *P. nigromaculatus* and *N. quadranus*, although their types and abundances varied seasonally and interspecifically. Overall, this study highlights seasonal dynamics and host-specific variations in amphibian cutaneous fungal ecology and identifies potential Bd-inhibiting fungal taxa.

## 1. Introduction

Amphibians face multiple global threats, with emerging fungal pathogens being particularly prominent [1]. The pathogenic chytrid fungi *Batrachochytrium dendrobatidis* (Bd) and *B. salamandrivorans* (Bsal), which are responsible for chytridiomycosis, infect amphibian skin, disrupt the osmoregulatory balance, and cause mortality [2,3]. These pathogens have contributed to population declines in over 500 amphibian species worldwide [1]. Furthermore, ranavirosis is also an emerging viral infectious disease that has been documented to cause mass mortality in amphibians [4,5]. Seasonal aggregations in amphibians represent one of the key factors modulating disease risk. For instance, during the spring breeding season, they exhibit pronounced high-density aggregations. This significantly increases opportunities for direct skin-to-skin contact among individuals and leads to the rapid accumulation of pathogens and host-derived organic matter in confined aquatic environments, creating localized infection hotspots [6,7]. Although chytridiomycosis and ranavirosis typically exhibit high mortality rates, interspecific variation in resistance to their pathogen infections exists, which is partially attributable to skin microbial communities [8].

The amphibian skin and its microbial consortium constitute the primary defense against pathogen invasion [9,10,11]. Skin-associated bacterial communities protect against Bd and Bsal infections by producing specific bacterial metabolites and volatile compounds [12,13,14]. The experimental depletion of skin bacteria increases susceptibility to chytridiomycosis in some amphibians, whereas probiotic supplementation demonstrates protective effects [15,16,17]. The role of cutaneous bacterial communities in modulating host immune defense against pathogens is being increasingly recognized. However, research on amphibian cutaneous fungal communities and their interactions with host health remains limited [3,18]. The structure and composition of skin bacterial and fungal communities were significantly associated with the status and intensity of Bd infection [19]. Furthermore, the interactions within microbial communities also influence host defenses against pathogens [19].

Fungal components in animal-associated microbiota, although rare in the biosphere (less than 0.1% of microbial cells), show higher diversity than expected, including novel lineages [18,20]. In eukaryotes, fungi are larger than bacteria and have complex cellular structures and functions, multiple organelles, and larger genomes. Therefore, mycobiomes may offer unique genetic and metabolic features to animal hosts and influence the overall properties of the microbiota [21,22]. Notably, non-Bd fungi on amphibian skin have been implicated in disease resistance [3]. Additionally, fungal communities on amphibian skin may harbor other pathogenic fungi, including chromomycosis, basidiobolomycosis, and mucormycosis [23].

Temporal or seasonal variations in cutaneous microbiota may be another critical factor in disease dynamics. In our previous research, we analyzed seasonal shifts in amphibian skin bacterial communities and interspecific differences and predicted the corresponding changes in their anti-Bd activity [24]. In this study, we focused on *Pelophylax nigromaculatus* (Hallowell, 1861) and *Nanorana quadranus* (Liu, Hu & Yang, 1960) from Huangbaiyuan in the Qinling Mountains and shifted our emphasis to fungal communities. We aimed to address the following questions: (1) how do the composition and diversity of amphibian cutaneous mycobiomes vary with seasonal cycles and host species? (2) Do Bd-inhibiting fungi exist in mycobiomes?

To answer these questions, we conducted seasonal sampling of skin mycobiomes from both species, analyzed their diversity and structure, and assessed whether amplicon sequence variants (ASVs) exhibited anti-Bd properties. Our findings enhance our understanding of the cutaneous microbial and mycobiome ecology in Qinling anurans. Concurrently, this study explored the defensive roles of symbiotic mycobiomes and provided actionable data to inform contemporary amphibian conservation strategies.

## 2. Materials and Methods

### 2.1. Field Specimen Acquisition and Processing Protocol

We conducted non-lethal sampling of two anuran species, *P. nigromaculatus* (*n* = 20; aquatic and quiet-water type) and *N. quadranus* (*n* = 12; aquatic and running-water type), within Huangbaiyuan Town (Qinling Mountains, Baoji, China) during three consecutive annual growth cycles (April–September 2023). Specimen procurement was avoided during the hidden periods corresponding to metabolic dormancy. Target species selection followed the IUCN population viability assessments to ensure cohort representativeness. The sampling coordinates and times are listed in Table 1.

Sterile polyethylene gloves (changed between individuals) were used to manually capture the frogs. To reduce transient environmental microbiota, each specimen was subjected to three sequential rinses with purified water prior to swabbing. Epidermal microbiota was collected by firmly swabbing 30 times across standardized body regions (dorsal, ventral, and limb surfaces) using sterile skin swabs. Swab heads were immediately transferred to 2 mL of DNA storage solution (consisted of Tris, EDTA-2Na, and NaCl; Shanghai Langfu Industrial, Shanghai, China) and stored at 4 °C. Collected samples were transported on dry ice to Beijing Biomarker Technologies for sequencing.

### 2.2. DNA Extraction and Sequencing

Total genomic DNA was extracted using magnetic bead-based purification (TGuide S96 Soil/Epithelial DNA Kit, Tiangen, Beijing, China) with enzymatic pretreatment (lysozyme, 10 mg/mL; 37 °C/1 h). Amplification targeted the ITS (internal transcribed spacer) domain of fungal organisms using primers (ITS1F: 5′-CTTGGTCATTTAGAGGAAGTAA-3′; ITS2R: 5′-GCTGCGTTCTTCATCGATGC-3′) under the following thermocycling conditions: 95 °C/3 min of initial denaturation; 20 cycles of 95 °C/30 s, 58 °C/30 s, and 72 °C/45 s; and a final extension at 72 °C/10 min. The polymerase chain reaction (PCR) products were quantified through agarose gel electrophoresis and purified using a DNA purification kit (Omega, Norcross, GA, USA). Purified libraries were subjected to paired-end sequencing (PE250) using Illumina NovaSeq 6000 (Illumina Inc., San Diego, CA, USA).

### 2.3. Bioinformatic Processing Pipeline

The sequence analysis pipeline was executed using the BMK Cloud platform. The initial raw data preprocessing involved quality-based nucleotide filtering using Trimmomatic (v0.33) [25], followed by primer sequence detection and elimination using Cutadapt (v1.9.1) [26]. Subsequent quality refinement employed the filterAndTrim module with a maximum expected error threshold set to 2 (EE = Σ10^(−Q/10)^), retaining default values for other parameters. The workflow progressed through three computational phases as follows: (1) error profile modeling through the learnErrors algorithm, (2) sequence noise reduction using the DADA2 implementation, and (3) paired-end read assembly through mergePairs with optimized parameters (minimum overlap: 18 bp; maximum mismatches: 20% of overlap length). Final chimera detection and elimination were achieved using the removeBimeraDenovo method in consensus mode [27,28,29].

### 2.4. Fungal Community Profiling

Amplicon sequence variants (ASVs) were generated from quality-filtered reads using DADA2, excluding low-abundance features (total counts < 2 across all specimens) [29]. Taxonomic assignment was performed in QIIME2 by aligning the ASVs against the SILVA reference database (v138.1) using a naive Bayesian classifier with a 70% confidence cutoff [30].

Alpha diversity metrics (Shannon, Simpson, Chao1, and ACE) were computed using QIIME2 pipelines and visualized using ggplot2 (v3.1.1). Interspecies variations in α-diversity were assessed using Wilcoxon rank-sum tests (significance threshold: *p* < 0.05). Beta diversity patterns were evaluated through binary Jaccard and Bray–Curtis distance metrics, with dimensionality reduction achieved through principal coordinate analysis (PCoA). A permutational multivariate analysis (PERMANOVA), using the Adonis method (Vegan package v2.3-0 in R v3.1.1), was used to quantify the between-group differences [31,32]. Hierarchical clustering patterns were determined using the UPGMA algorithm in Python 2 (ete3, v3.0.0b35). LEfSe biomarker discovery identified taxon-specific differential abundances using distinct LDA thresholds, with 4.5 for seasonal comparisons and 4.0 for host species differentiation [33].

To investigate the presence of Bd-inhibiting fungi in the samples, sequence alignments were performed between 30 known anti-Bd fungal ITS sequences and our ITS amplicon data. The 30 anti-Bd fungal ITS sequences, functionally validated by Kearns et al. (2017), inhibited either Bd strains 197 and 423 or both [3].

Specimens were categorized temporally as follows: *P. nigromaculatus* populations comprised SpringPn (*n* = 9), SummerPn (*n* = 6), and AutumnPn (*n* = 5) cohorts. *N. quadranus* samples unavailable in spring included the SummerNq (*n* = 6) and AutumnNq (*n* = 6) groups (Table 2).

## 3. Results

### 3.1. Overview of Cutaneous Fungal Communities

After sequencing, data filtering, and sequence assembly of the ITS amplicons from 32 frog skin samples, we obtained 2,723,044 sequences. These sequences were further processed and clustered into 8662 ASVs, primarily belonging to four phyla, namely Ascomycota (4569), Basidiomycota (1579), Mortierellomycota (294), and Chytridiomycota (330), with an additional 1441 ASVs remaining unclassified under fungi (unclassified_Fungi).

**Figure 1:**
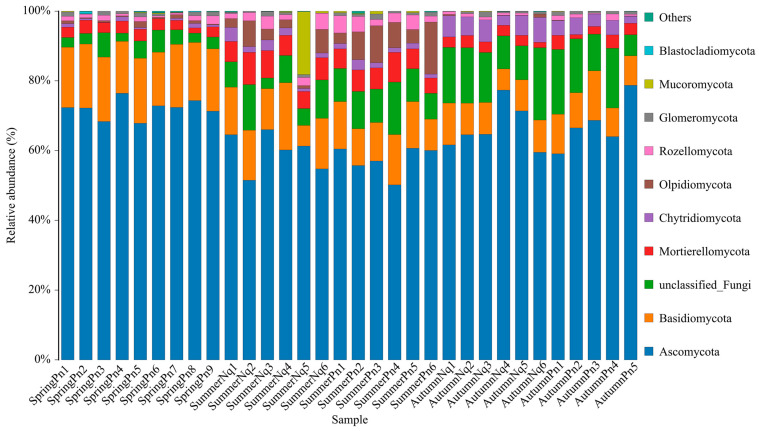
Histogram of skin fungi distribution at the phylum level. Different colors indicate different species; stacked columns are the top 10 taxa in relative abundance at each taxonomic level.

Table 2 and Figure 1 show the ASV counts per sample and the relative abundances of ASVs from each phylum across the samples. We identified 42 ASVs shared among all five groups, which were classified as Ascomycota (30), Basidiomycota (7), Mortierellomycota (3), and unclassified_Fungi (2) (Supplementary Figure S1).

### 3.2. Seasonal Variation Influenced Fungal Communities

The SpringPn, SummerPn, and AutumnPn groups shared 58 ASVs, accounting for 1.12% of the total across both datasets (Supplementary Figure S2). The SummerNq and AutumnNq groups shared 177 ASVs (3.81%) (Supplementary Figure S3).

**Figure 2:**
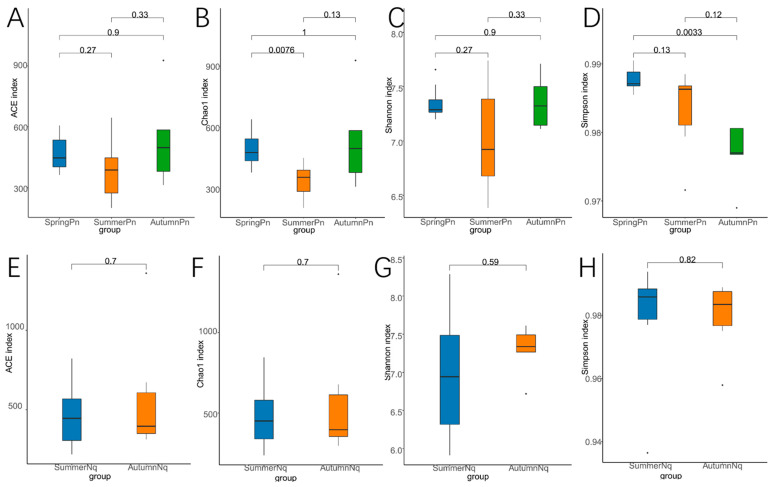
Box plot of variation in alpha diversity indices for skin fungal communities on *Pelophylax nigromaculatus* across three seasons (**A**–**D**) and on *Nanorana quadranus* across two seasons (**E**–**H**). The horizontal coordinates are the group names, and the vertical coordinates are the values of the corresponding alpha diversity indices. (**A**,**E**) Wilcoxon test of Chao1 index; (**B**,**F**) Wilcoxon test of ACE index; (**C**,**G**) Wilcoxon test of Shannon index; (**D**,**H**) Wilcoxon test of Simpson index.

Alpha diversity analysis revealed significant seasonal variations in the species richness and diversity patterns of the fungal communities in *P. nigromaculatus* (Table 2, Figure 2A–D). The Chao1 index indicated that the estimated species richness in the SpringPn group was significantly higher than that in the SummerPn group (*p* < 0.01), whereas no intergroup differences were detected in the ACE index (*p* > 0.05). In terms of diversity, the Shannon index showed no significant differences among the groups (*p* > 0.05); however, the Simpson index demonstrated a significant divergence between the SpringPn and AutumnPn groups (*p* < 0.01). In contrast, no significant seasonal differences in species richness or diversity were observed for *N. quadranus* (Table 2, Figure 2E–H).

**Figure 3:**
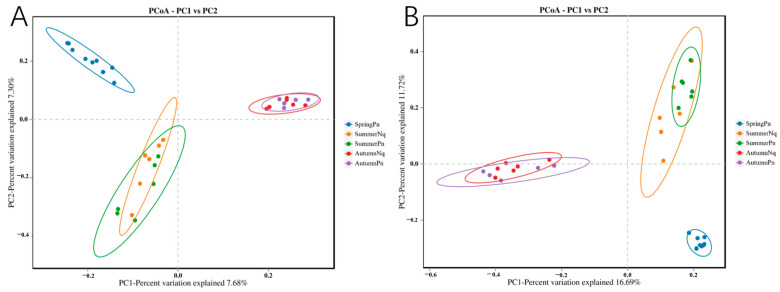
Principal coordinate analysis of beta diversity in skin fungal communities on all groups. (**A**) Binary Jaccard distance; (**B**) Bray–Curtis distance. Each point represents the cutaneous fungal community of an individual sample.

Using the PCoA of binary Jaccard and Bray–Curtis distance metrics, we identified seasonal differences in the species composition of skin fungal communities. On the PC1 axis, the fungal communities of SpringPn and SummerPn exhibited a partial overlap in their clustering positions, whereas AutumnPn showed considerable divergence from both SpringPn and SummerPn. On the PC2 axis, the skin fungal communities of the AutumnPn group clustered centrally, with SpringPn and SummerPn positioned above and below this central cluster, respectively, indicating distinct differences among the three seasonal groups (Figure 3A,B). For *N. quadranus*, the PC1 axis was the primary factor driving significant differences between SummerNq and AutumnNq (Figure 3A,B). The results from the PERMANOVA (*p* = 0.001, treatments 1 and 2 in Table 3 and Table 4) confirmed seasonal differences in fungal communities between the two frog species.

The skin fungi of the SpringPn, SummerPn, and AutumnPn groups shared highly similar compositions of the top five phyla, although their relative abundances differed (Figure 1, Table 5). Similar patterns were observed for *N. quadranus* across all seasons (Figure 1, Table 5). Notably, the phylum Ascomycota exhibited overwhelmingly dominant relative abundance in all the groups.

**Figure 4:**
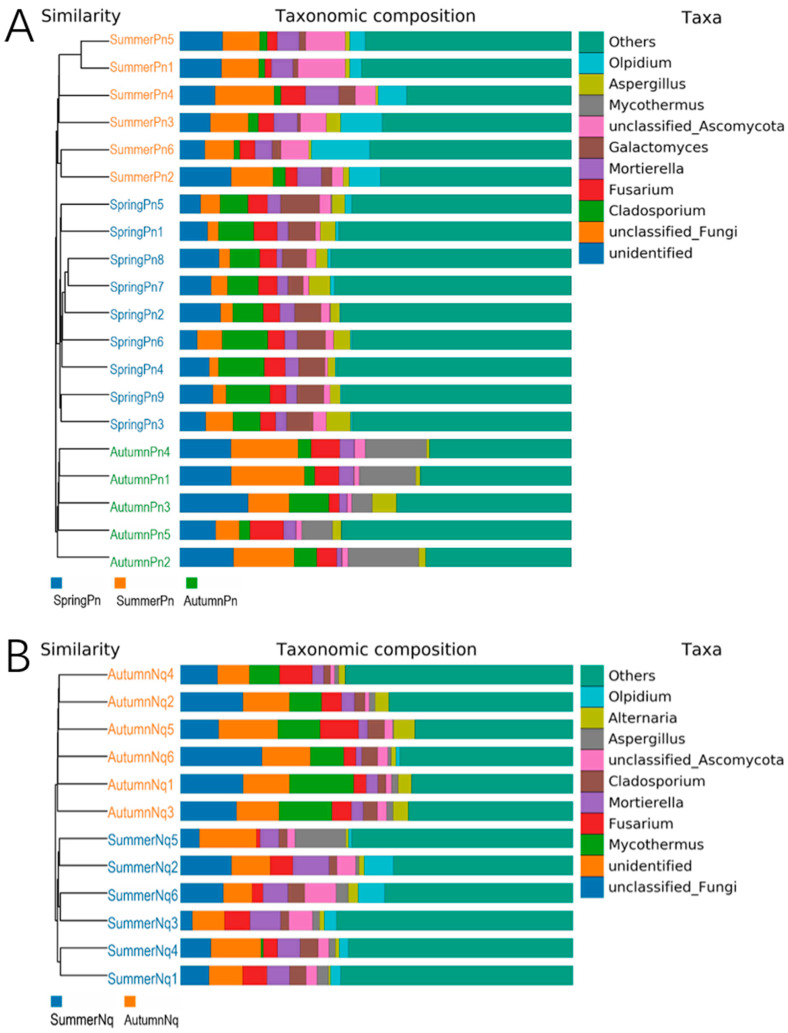
UPGMA clustering tree (binary Jaccard distance) combined with species distribution histogram (genus level) for *P. nigromaculatus* in three seasons (**A**) and *N. quadranus* in two seasons (**B**). Clustering tree on the left; species distribution histogram on the right.

The UPGMA clustering tree (binary Jaccard distance) combined with the species distribution histograms (genus level) showed that samples collected during the same season were more similar in terms of species composition (Figure 4). Species composition and relative abundance significantly varied between the seasons (Figure 4A,B and Table 6). One unidentified genus was present in samples from both frogs in different seasons.

**Figure 5:**
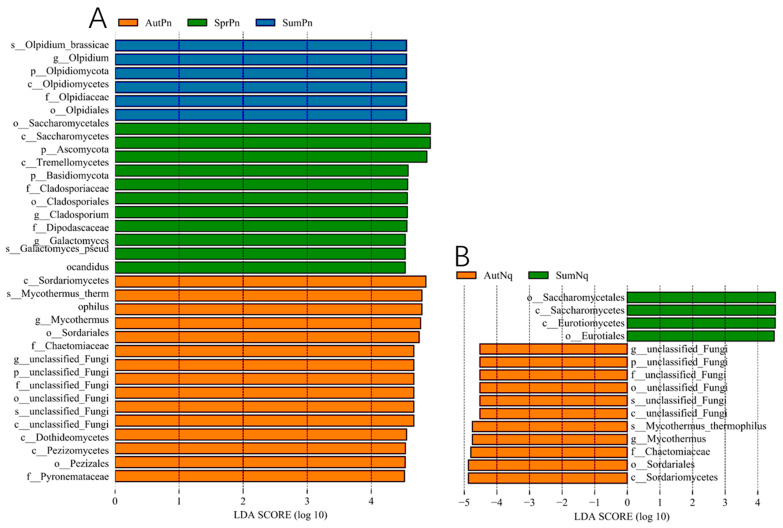
Histogram of the distribution of LDA values (LDA score of 4.5) comparing *P. nigromaculatus* in three seasons (**A**) and *N. quadranus* in two seasons (**B**). The vertical axis represents the taxonomic units exhibiting significant differences between the groups, while the horizontal axis displays bar graphs illustrating the logarithmic scores of LDA for each respective taxonomic unit.

Next, LEfSe (LDA score of 4.5) analyses of the fungal populations with intergroup differences in relative abundance revealed the presence of multiple biomarkers (Figure 5). At the genus level, the most abundant biomarker in SpringPn was *Olpidium*, while those in SummerPn were *Cladosporium* and *Galactomyces* and those in AutumnPn were *Mycothermus* and *unclassified_Fungi* (Figure 5A). The most abundant biomarkers in SummerNq were *unclassified_Fungi* and *Mycothermus* (Figure 5B). The persistence of the unclassified_Fungi genus across seasons and hosts may represent an understudied symbiotic lineage with generalized stress tolerance.

### 3.3. Effect of Host Species on Cutaneous Fungal Communities

A Venn diagram analysis of cutaneous fungal ASVs demonstrated that 468 ASVs (17.57% of the total ASVs) were shared between the SummerPn and SummerNq groups, whereas 487 ASVs (10.66% of all ASVs) overlapped in the autumn groups (Supplementary Figures S4 and S5).

**Figure 6:**
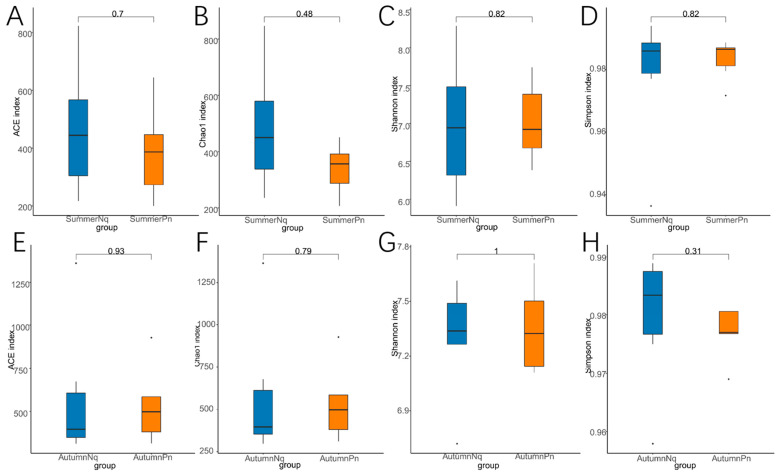
Box plot of variation in alpha diversity indices for skin fungal communities on *P. nigromaculatus* and *N. quadranus* in the same seasons, including summer (**A**–**D**) and autumn (**E**–**H**). The horizontal coordinates are the group names, and the vertical coordinates are the values of the corresponding alpha diversity indices. (**A**,**E**) Wilcoxon test of Chao1 index; (**B**,**F**) Wilcoxon test of ACE index; (**C**,**G**) Wilcoxon test of Shannon index; (**D**,**H**) Wilcoxon test of Simpson index.

The alpha diversity metrics (Chao1, ACE, Shannon, and Simpson) revealed no significant interspecific differences in species richness or diversity within the same season (*p* > 0.05, Figure 6A–H), suggesting comparable overall fungal diversity despite host species divergence.

A beta diversity analysis further supported this pattern. The PCoA revealed an extensive overlap in the confidence ellipses formed by the sample groups of the two frog species within the same season (Figure 3A,B). The PERMANOVA (*p* > 0.05, treatments 3 and 4 in Table 3 and Table 4) confirmed no statistically significant interspecific differences in beta diversity during the same seasonal period.

**Figure 7:**
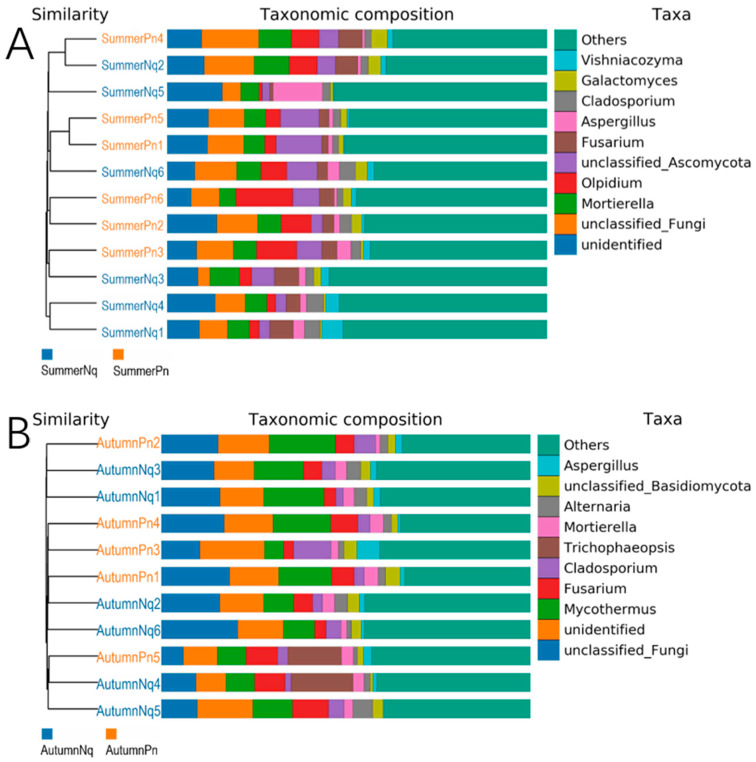
UPGMA clustering tree (binary Jaccard distance) combined with species distribution histogram (genus level) for *P. nigromaculatus* and *N. quadranus* in the same seasons, including summer (**A**) and autumn (**B**).

The top five phyla were identical among different species across the same season, although their relative abundances differed (Figure 1; Table 5). At the genus level, four of the five dominant genera interspecifically overlapped; however, their abundance distributions exhibited host-specific modulations (Table 6; Figure 7A,B). The UPGMA clustering tree (Figure 7A,B) verified that these skin samples shared similar microbial species compositions within the same season.

**Figure 8:**
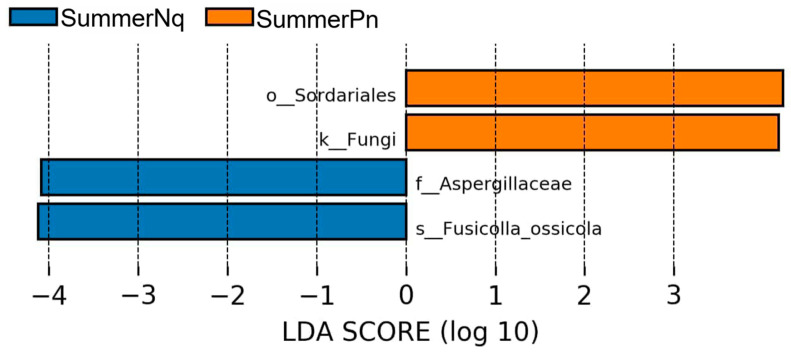
Histogram of the distribution of LDA values (LDA score of 4.0) comparing *P. nigromaculatus* and *N. quadranus* in the same seasons. The vertical axis represents the taxonomic units exhibiting significant differences between the groups, while the horizontal axis displays bar graphs illustrating the logarithmic scores of LDA for each respective taxonomic unit.

When the LDA score was set to 4.0, the SummerPn and SummerNq groups had one biomarker each, namely *Fusicolla ossicola* (species level) and Sordariales (order level) (Figure 8), respectively. None of the frog species exhibited biomarkers in autumn.

### 3.4. Presence of Bd-Inhibiting Fungi Across Different Groups

A comparative analysis of the 30 known anti-Bd fungal ITS sequences and ITS amplicon data obtained in this study revealed 10, 4, 7, 12, and 7 anti-Bd ASVs in the SpringPn, SummerPn, AutumnPn, SummerNq, and AutumnNq groups, respectively. Moreover, the types and abundance of anti-Bd ASVs vary across seasons and species. For instance, a *Trichoderma*-assigned ASV (ASV10874) exhibited strict seasonal specificity, being present in the SummerPn and SummerNq groups but absent in both spring and autumn samples.

## 4. Discussion

Seasonal fluctuations and host-specific factors can influence pathogen resistance by affecting amphibian skin bacterial communities [11,24,34,35,36]. In this study, we investigated the effects of seasonal fluctuations and host-specific factors on skin mycobiomes by analyzing the skin fungal communities of two Qinling anurans, *P. nigromaculatus* and *N. quadranus*, across distinct seasonal periods. Our results demonstrated that seasonal variation exerted a significantly stronger influence on skin mycobiome structures than the host species. This pattern was consistently observed across the metrics of alpha diversity (Chao1 and Simpson indices in *P. nigromaculatus*), beta diversity, species composition and abundance, and biomarkers.

In *P. nigromaculatus*, the discrepancy between the Chao1 and ACE indices suggests that the expansion of singletons/doubletons (species observed only once or twice) in the SpringPn group may have resulted from short-term environmental filtering. However, this transient effect did not significantly alter the broader low-abundance fungal community. Regarding the contrasting patterns of the Shannon and Simpson indices, the emergence of two new dominant genera in the AutumnPn group (summed relative abundance = 25.79%) probably drove the significance of the Simpson index, whereas the non-significant Shannon index reflects compensatory contributions from rare species that maintain the overall diversity equilibrium.

In terms of fungal community composition, the intergroup consistency was significantly higher at the phylum level than at the genus level. Ascomycota dominated overwhelmingly, followed by Basidiomycota, both of which represent dominant phyla associated with animal-associated fungal communities [18,20]. Seasonal distribution shifts were observed in the cutaneous mycobiome of *P. nigromaculatus* in Rozellomycota, Olpidiomycota, and Chytridiomycota (spring, summer, and autumn), whereas *N. quadranus* showed seasonal variations in Olpidiomycota and Chytridiomycota (summer and autumn) (Table 5). This phenomenon likely reflects a dynamic equilibrium shaped by environmental factors, the host physiological state, and microbial interactions. The three following mechanistic hypotheses have been proposed: (1) elevated summer temperatures may suppress the growth of temperature-sensitive Chytridiomycota fungi, such as Bd, while providing competitive advantages to thermotolerant taxa, such as Olpidiomycota. (2) Amphibian skin secretions, including antimicrobial peptides (AMPs) and mucoproteins, undergo seasonal compositional changes, potentially altering fungal colonization dynamics. (3) Seasonal fluctuations in cutaneous bacterial communities may indirectly reshape the mycobiome structure. For instance, the increased summer abundance of anti-Bd bacterial metabolites, such as violacein, can inhibit Chytridiomycota while facilitating the proliferation of other fungal phyla [12,14,24].

Although Chytridiomycota (a phylum containing Bd and Bsal) was detected in both the AutumnPn and AutumnNq groups, no representatives of the genus *Batrachochytrium* were identified at the genus level. This genus remained undetected in all samples, which was consistent with our previous PCR-based screening results [24]. Although no *Batrachochytrium* was detected, the persistent presence of non-pathogenic Chytridiomycota ASVs across seasons suggests these commensals may occupy ecological niches otherwise exploited by Bd or Bsal.

Studies have demonstrated that the inhibitory capacity of amphibian cutaneous mycobiomes against Bd significantly surpasses that of co-occurring bacterial communities [3]. However, our analysis was confined to the comparative alignment of 30 anti-Bd fungal ITS reference sequences. The absence of a comprehensive database encompassing all documented and undocumented Bd-inhibiting fungal taxa limited the definitive quantification of anti-Bd fungal ASVs and their relative abundances across different groups. However, the types and abundances of known anti-Bd fungal ASVs vary with the season and species. It is crucial to emphasize that these observed data cannot be extrapolated to the Bd-inhibitory capability of cutaneous mycobiomes, as there is still a large number of fungi with unknown resistance to Bd.

In probiotic applications, while bacterial metabolites likely contribute to chytridiomycosis suppression in summer, fungal–bacterial interactions may extend beyond antibiosis. *Penicillium expansum* could facilitate bacterial biofilm formation through extracellular polymeric substance production, potentially enhancing collective anti-pathogen functions [37]. Such cross-kingdom synergies warrant metatranscriptomic validation. Additionally, bacterial probiotics may stress amphibian hosts and inhibit antimicrobial peptide responses, limiting their potential for long-term colonization [3]. In contrast, fungal probiotics, such as *P. expansum*, may not induce host stress responses or inhibit host antimicrobial peptide responses compared with bacterial probiotics, although the generality of this finding among hosts and potential probiotics is unknown [3]. This finding suggests that fungal probiotics may be a more suitable long-term treatment strategy for amphibian chytridiomycosis.

Notably, under climate warming scenarios, the observed summer decline of temperature-sensitive Chytridiomycota may intensify. Paradoxically, this could favor thermotolerant pathogens like Ranavirus—which caused stress in host species at higher temperture—necessitating vigilance toward multi-pathogen threats during thermal extremes [4].

## 5. Conclusions

Our study demonstrated that seasonal variation has a significantly stronger effect on cutaneous mycobiome structures than host species in amphibians. These effects were pronounced in changes to α-diversity, β-diversity, species composition, abundance, and biomarkers. Our study also confirmed the presence of anti-Bd fungi in the skin fungal communities of *P. nigromaculatus* and *N. quadranus* in the Qinling Mountains, although their types and abundances varied across seasons and species. In conclusion, our findings emphasize the dual effects of seasonal variation and host species on amphibian skin fungal biomes. These findings provide foundational knowledge for future studies on host–fungi interactions and structure–function relationships within the amphibian cutaneous mycobiome, with important implications for understanding symbiotic ecology and chytridiomycosis resilience. These findings, along with those of related studies, can potentially benefit research on disease etiology and controlling cutaneous microbes in other wildlife hosts.

## Figures and Tables

**Table 1:** Sample information of anuran species included in this study.

Species	Sampling Time	Sample Size	Location	Elevation (m)	Site Description	IUCN Red List Category
*Pelophylax* *nigromaculatus*	25 April	9 (F, 4; M, 5)	33.70964668° N 107.39150169° E	989	paddy fields	LC
	17 July	6 (F, 3; M, 3)				
	19 September	5 (F, 2; M, 3)				
*Nanorana* *quadranus*	25 April	\	33.70561351° N 107.38986328° E	988	stream	NT
	17 July	6 (F, 3; M, 3)				
	19 September	6 (F, 3; M, 3)				

Abbreviations: IUCN, International Union for Conservation of Nature; LC, least concern; NT, near threatened. Data source: IUCN Red List (version 2024.3; www.iucnredlist.org (accessed 20 June 2025)).

**Table 2:** Number of amplicon sequence variants (ASVs) and alpha diversity indices in each sample.

Group	Sample	ASV Number	Abundance Index		Diversity Index	
			Chao1	ACE	Shannon	Simpson
SpringPn	SpringPn1	376	439.0	402.528	7.2642	0.9869
	SpringPn2	572	639.0286	606.4556	7.5198	0.9903
	SpringPn3	326	380.0769	362.7632	7.379	0.9905
	SpringPn4	403	513.55	534.5982	7.3516	0.9858
	SpringPn5	366	415.4	399.5598	7.276	0.9871
	SpringPn6	387	477.2308	477.0094	7.2877	0.9868
	SpringPn7	512	543.9545	544.9311	7.6572	0.9888
	SpringPn8	366	555.1	441.9499	7.2505	0.988
	SpringPn9	376	437.1071	446.5383	7.2007	0.9855
SummerPn	SummerPn1	366	400.0	414.5363	7.5274	0.9865
	SummerPn2	266	346.5714	359.1739	6.8807	0.9861
	SummerPn3	173	207.0	200.1321	6.609	0.9794
	SummerPn4	226	366.25	644.7811	6.9626	0.9869
	SummerPn5	431	451.0	457.6898	7.7415	0.9885
	SummerPn6	183	268.0	244.8576	6.383	0.9716
AutumnPn	AutumnPn1	584	585.0	584.7134	7.7097	0.9806
	AutumnPn2	310	310.5	312.8443	7.144	0.9768
	AutumnPn3	380	380.0	380.3383	7.1098	0.9806
	AutumnPn4	496	497.0	497.0556	7.5015	0.977
	AutumnPn5	927	927.0	927.0	7.3232	0.969
SummerNq	SummerNq1	486	561.6	541.561	7.5384	0.9877
	SummerNq2	287	337.6	347.6141	7.3232	0.9886
	SummerNq3	177	235.6667	217.6583	6.235	0.9769
	SummerNq4	815	846.5	823.1168	8.2885	0.9938
	SummerNq5	546	585.1364	575.9615	5.9111	0.9364
	SummerNq6	244	338.0909	290.6159	6.5623	0.9839
AutumnNq	AutumnNq1	372	375.0	382.08	7.2666	0.975
	AutumnNq2	401	416.0	408.88	7.6133	0.9883
	AutumnNq3	282	296.0	311.5464	7.263	0.9851
	AutumnNq4	668	678.0	672.7716	6.7187	0.9579
	AutumnNq5	1358	1365.0909	1360.4544	7.5163	0.9817
	AutumnNq6	319	345.25	337.4353	7.4075	0.9889

**Table 3:** Summary of PERMANOVA models (binary Jaccard distance) of beta diversity for fungal communities on frog skin. Effects on variation due to season (treatment 1 and treatment 2) and species (treatment 3 and treatment 4) are considered. Significant results are marked with **.

Treat	Variables	Degrees of Freedom	Sums of Squares	Mean Squares	F. Model	R^2^	*p*
1	*P. nigromaculatus* in different seasons	2	1.670135	0.835068	2.068017	0.195686	0.001 **
2	*N. quadranus* in different seasons	1	0.698502	0.698502	1.604157	0.138240	0.001 **
3	*P. nigromaculatus* and *N. quadranus* in summer	1	0.448963	0.448963	1.064348	0.096196	0.342
4	*P. nigromaculatus* and *N. quadranus* in autumn	1	0.435276	0.435276	1.000984	0.100089	0.399

**Table 4:** Summary of PERMANOVA models (Bray–Curtis distance) of beta diversity for fungal communities on frog skin. Effects on variation due to season (treatment 1 and treatment 2) and species (treatment 3 and treatment 4) are considered. Significant results are marked with **.

Treat	Variables	Degrees of Freedom	Sums of Squares	Mean Squares	F. Model	R^2^	*p*
1	*P. nigromaculatus* in different seasons	2	2.474165	1.237082	4.260942	0.333905	0.001 **
2	*N. quadranus* in different seasons	1	1.023472	1.023472	2.893324	0.224405	0.001 **
3	*P. nigromaculatus* and *N. quadranus* in summer	1	0.41986	0.41986	1.144997	0.102736	0.232
4	*P. nigromaculatus* and *N. quadranus* in autumn	1	0.262063	0.262063	0.868163	0.087976	0.625

**Table 5:** The composition and relative abundance of the top five phyla in each group. “\”means the phylum of the corresponding group did not appear in the top five.

Phylum	SpringPn	SummerPn	AutumnPn	SummerNq	AutumnNq
Ascomycota	72.02 ± 0.89%	57.37 ± 1.66%	67.42 ± 3.25%	59.72 ± 2.30%	65.53 ± 2.71%
Basidiomycota	17.29 ± 0.45%	11.97 ± 0.87%	10.45 ± 1.10%	13.24 ± 1.78%	9.06 ± 0.76%
unclassified_Fungi	4.08 ± 0.56%	10.28 ± 1.05%	13.54 ± 2.32%	7.85 ± 1.52%	14.38 ± 1.74%
Mortierellomycota	2.99 ± 0.22%	6.09 ± 0.55%	2.92 ± 0.53%	6.67 ± 0.65%	2.86 ± 0.27%
Chytridiomycota	\	\	3.75 ± 0.47%	\	5.54 ± 0.61%
Olpidiomycota	\	7.97 ± 1.80%	\	3.84 ± 1.08%	\
Rozellomycota	1.16 ± 0.21%	\	\	\	\

**Table 6:** The composition and relative abundance of the top five genera in each group. “\”means the genus of corresponding group did not appear in the top five.

Genus	SpringPn	SummerPn	AutumnPn	SummerNq	AutumnNq
*Cladosporium*	9.02 ± 0.66%	\	4.93 ± 1.41%	\	\
unidentified	7.54 ± 0.66%	9.66 ± 0.99%	13.33 ± 1.32%	10.18 ± 1.16%	11.64 ± 0.92%
*Galactomyces*	6.79 ± 0.51%	\	\	\	\
*Fusarium*	4.67 ± 0.23%	\	5.97 ± 1.00%	4.28 ± 0.91%	5.74 ± 1.12%
*unclassified_Saccharomycetales*	4.16 ± 0.26%	\	\	\	\
*unclassified_Fungi*	\	10.28 ± 1.05%	13.54 ± 2.32%	7.85 ± 1.52%	14.38 ± 1.74%
*Olpidium*	\	7.97 ± 1.79%	\	\	\
*unclassified_Ascomycota*	\	7.32 ± 1.36%	\	4.39 ± 0.95%	\
*Mortierella*	\	6.05 ± 0.56%	\	6.66 ± 0.66%	\
*Mycothermus*	\	\	12.25 ± 2.43%	\	10.83 ± 1.41%
*Alternaria*	\	\	\	\	3.19 ± 0.61%

## Data Availability

The original contributions presented in this study are included in the article/Supplementary Materials. Further inquiries can be directed to the corresponding author.

## Supplementary Materials

The following supporting information can be downloaded at: https://www.mdpi.com/article/10.3390/jof11070473/s1, Figure S1: Venn diagram presenting number of shared and unique ASVs between each group of all samples; Figure S2: Venn diagram presenting number of shared and unique ASVs between each group of *Pelophylax nigromaculatus*; Figure S3: Venn diagram presenting number of shared and unique ASVs between each group of *Nanorana quadranus*; Figure S4: Venn diagram presenting number of shared and unique ASVs between *P. nigromaculatus* and *N. quadranus* in summer; Figure S5: Venn diagram presenting number of shared and unique ASVs between *P. nigromaculatus* and *N. quadranus* in autumn.
