# Supplementary material for: Seasonality Has Greater Influence on Amphibian Cutaneous Mycobiome than Host Species
Source: J Fungi (Basel). 2025 Jun 22;11(7):473. doi: 10.3390/jof11070473 (PMC12295963; doi:10.3390/jof11070473)
Supplement: Supplementary file 1 [file jof-11-00473-s001.zip › jof-3675068-supplementary.pdf]

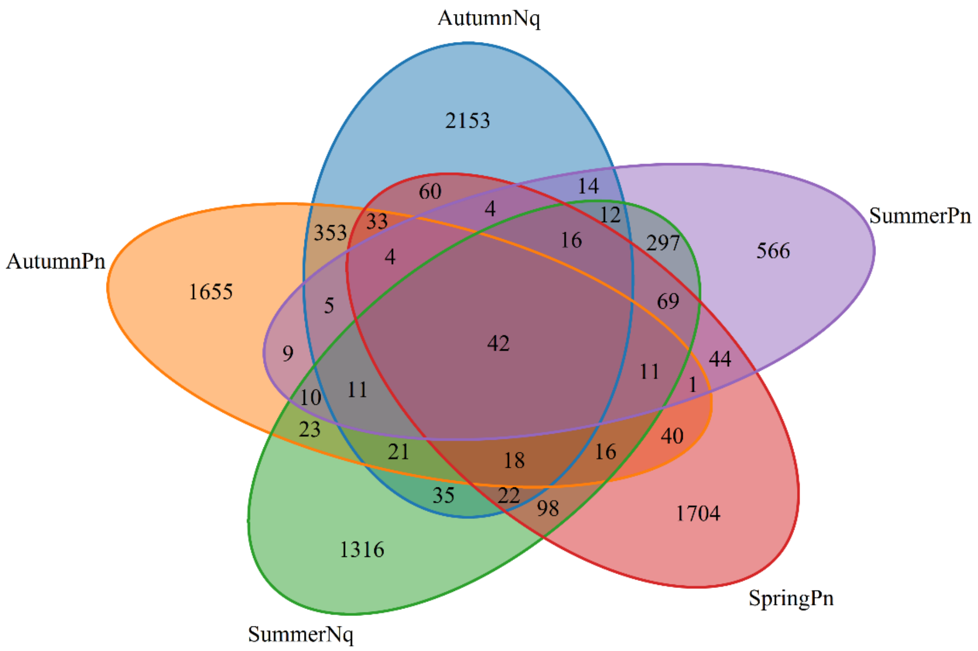

Supplementary Figure S1 Venn diagram presenting number of shared, unique ASVs between each groups of all samples.

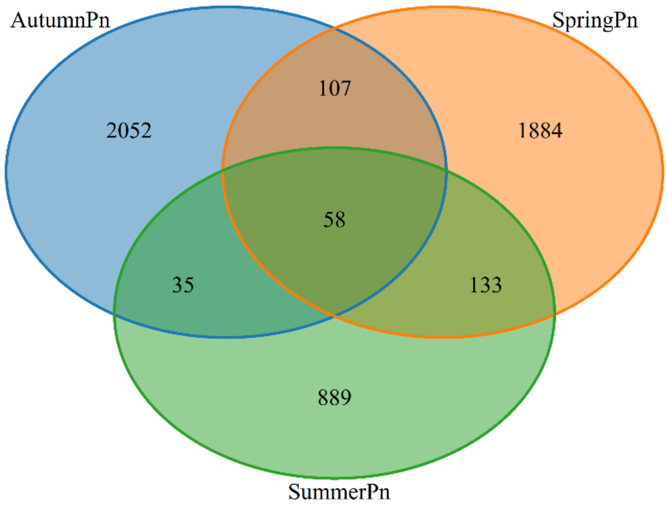

Supplementary Figure S2 Venn diagram presenting number of shared, unique ASVs between each groups of *Pelophylax nigromaculatus*.

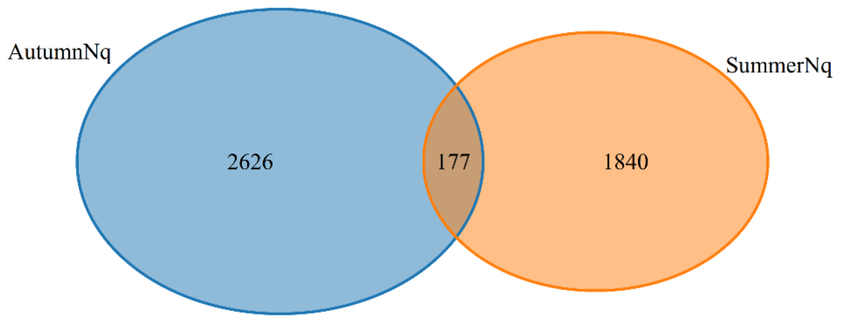

Supplementary Figure S3 Venn diagram presenting number of shared, unique ASVs between each groups of *Nanorana quadranus*.

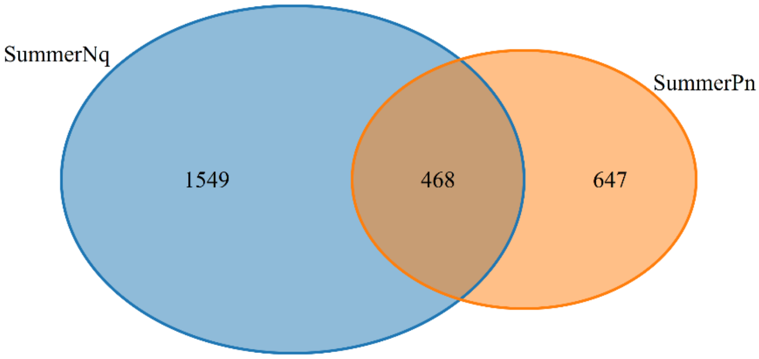

Supplementary Figure S4 Venn diagram presenting number of shared, unique ASVs between *P. nigromaculatus* and *N. quadranus* in summer.

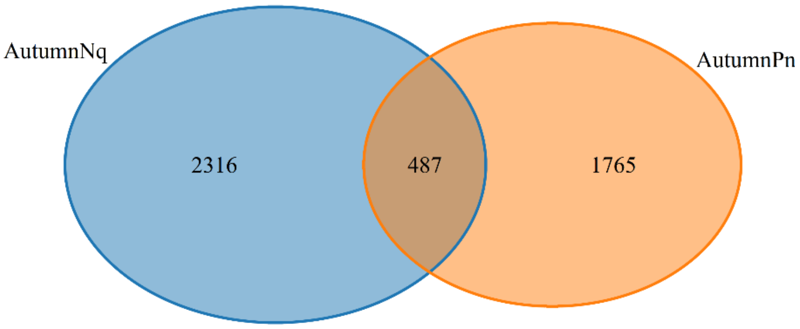

Supplementary Figure S5 Venn diagram presenting number of shared, unique ASVs between *P. nigromaculatus* and *N. quadranus* in autumn.
